# Transcriptional signatures of regulatory and toxic responses to benzo-[a]-pyrene exposure

**DOI:** 10.1186/1471-2164-12-502

**Published:** 2011-10-13

**Authors:** Jacob J Michaelson, Saskia Trump, Susanne Rudzok, Carolin Gräbsch, Danielle J Madureira, Franziska Dautel, Juliane Mai, Sabine Attinger, Kristin Schirmer, Martin von Bergen, Irina Lehmann, Andreas Beyer

**Affiliations:** 1 Cellular Networks and Systems Biology, Biotechnology Center, TU Dresden, Dresden, Germany; 2 Dept. of Environmental Immunology, UFZ, Helmholtz Center for Environmental Research, Leipzig, Germany; 3 EAWAG Aquatic Research, ETH Swiss Federal Institute of Technology, Zürich, Switzerland; 4 Dept. of Proteomics, UFZ, Helmholtz Center for Environmental Research, Leipzig, Germany; 5 Dept. of Computational Hydro Systems, UFZ, Helmholtz Center for Environmental Research, Leipzig, Germany; 6 EPF Lausanne, Lausanne, Switzerland; 7 Dept. of Metabolomics, UFZ, Helmholtz Center for Environmental Research, Leipzig, Germany

## Abstract

**Background:**

Small molecule ligands often have multiple effects on the transcriptional program of a cell: they trigger a receptor specific response and additional, indirect responses ("side effects"). Distinguishing those responses is important for understanding side effects of drugs and for elucidating molecular mechanisms of toxic chemicals.

**Results:**

We explored this problem by exposing cells to the environmental contaminant benzo-[a]-pyrene (B[a]P). B[a]P exposure activates the aryl hydrocarbon receptor (*Ahr*) and causes toxic stress resulting in transcriptional changes that are not regulated through *Ahr*. We sought to distinguish these two types of responses based on a time course of expression changes measured after B[a]P exposure. Using Random Forest machine learning we classified 81 primary *Ahr *responders and 1,308 genes regulated as side effects. Subsequent weighted clustering gave further insight into the connection between expression pattern, mode of regulation, and biological function. Finally, the accuracy of the predictions was supported through extensive experimental validation.

**Conclusion:**

Using a combination of machine learning followed by extensive experimental validation, we have further expanded the known catalog of genes regulated by the environmentally sensitive transcription factor *Ahr*. More broadly, this study presents a strategy for distinguishing receptor-dependent responses and side effects based on expression time courses.

## Background

Elucidating the transcriptional response of cells to xenobiotic compounds like drugs or environmental contaminants is of primary importance for understanding the physiological effects of such compounds. However, exposure to xenobiotic compounds often induces a complex transcriptional response comprised of specific (i.e. transcription factor (TF) activated programs) and unspecific regulatory mechanisms. Dissecting these responses and identifying the transcriptional profiles associated with each individual (sub-)effect is essential for explaining specific and possible side effects of drugs or for predicting toxic responses of environmental contaminants.

**Figure 1:**
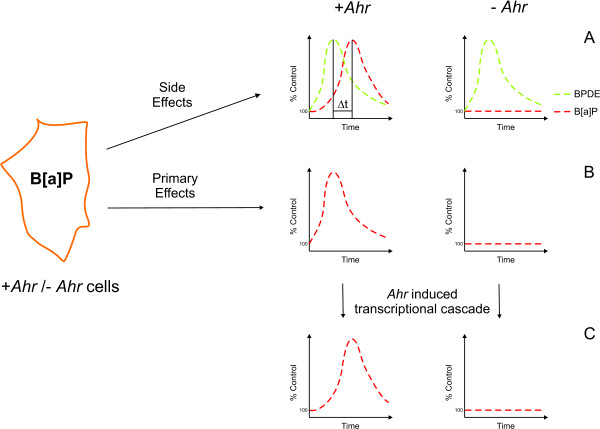
**Exposing cells to B[a]P provokes a complex intracellular response**. Exposing cells to B[a]P provokes a complex intracellular response. In *Ahr *expressing cells (+*Ahr*) B[a]P is metabolized, causing side effects due to its active metabolite BPDE. Since this metabolism is dependent on P450 enzymes that are activated by *Ahr*, side effects caused by B[a]P metabolism should only be detectable in +*Ahr*, while effects of direct exposure to BPDE should be independent of *Ahr *(A). Primary responders to *Ahr *are activated by B[a]P exposure only in +*Ahr *(B); these include other TFs. These TFs can in turn activate their target genes with a time lag compared to the primary *Ahr *response (C).

One of the most studied TFs involved in the response to environmental pollutants or xenobiotic compounds in general is the aryl hydrocarbon receptor (*Ahr*). The *Ahr *has been studied for decades mainly because of its critical role in xenobiotic-toxicity and carcinogenesis. In its inactive state, *Ahr *resides in the cytoplasm in a chaperone complex together with the X-associated protein 2 (*Xap2*, also known as *Aip*, *Ara9*) and heat-shock protein 90 (*Hsp90*). After ligand binding, the receptor translocates to the nucleus where it associates with its cofactor *Arnt *(*Ahr *nuclear translocator) yielding a competent TF. This heterodimer binds to a DNA binding motif called the xenobiotic response element (XRE), which functions as an enhancer in the regulatory domain of a wide range of genes commonly referred to as the *Ahr *gene battery [1,2]. Some of these genes, such as the cytochrome P450 enzyme *Cyp1a1*, NAD(P)H:quinine oxidoreductase (*Nqo1*), aldehyde dehydrogenase (*Aldh3a1*), UDP glucuronosyltransferase (*Ugt1a2*) and glutathione-S-transferase (*Gsta1*), are involved in Phase I/II metabolism. As previously mentioned, this activation of metabolizing enzymes through *Ahr *may lead to the formation of toxic metabolites of the activating ligand itself. This is particularly true for benzo-[a]-pyrene (B[a]P), a classical *Ahr *agonist. Only after the self-induced metabolism of this procarcinogen is the ultimate genotoxic metabolite anti-benzo-[a]-pyrene-trans-7, 8-dihydroxy-9, 10-epoxid (BPDE) formed. Several studies have examined the transcriptional effects of *Ahr *activation in different species and cell types [3-6]. However, deciphering the *Ahr*-specific transcriptional response is not a trivial task, considering that *Ahr *activation might trigger the activation of other TFs or the generation of toxic metabolites which will add side effects to the observed differential gene expression (Figure 1). Therefore, the overall transcriptional response directly related to *Ahr *binding is incompletely elucidated, and the number of well-defined *Ahr *specific genes still remains small.

One strategy to assess *Ahr*-dependence is to compare gene expression of cells or tissues that have the wild type *Ahr *with those of *Ahr*-null cells in a matched genetic background, as was shown by Tijet et al. [7]. In their study they compared the effect of 2,3,7,8-tetrachlorodibenzo-p-dioxin (TCDD) in *Ahr *+/+ and *Ahr *-/- mice after long term exposure. This experimental setup, as the authors themselves conceded, does not allow the discrimination of genes directly regulated through *Ahr *as a primary response and secondary, downstream effects: both classes would register as being differentially expressed. A time course design with early measurements has the potential to distinguish primary responders, which are likely to change first, from indirect responses that are likely to show up later.

In an elegant experimental setup, Hockley et al. [8,9] sought to separate the primary effects of *Ahr *activation from the side effect caused by the genotoxic metabolite BPDE. They compared the effects of B[a]P, BPDE and TCDD exposure in two different human cell lines. Unfortunately, the first time point they investigated was not until six hours after exposure. Considering that it was shown previously that *Ahr *translocation and nascent transcription is already induced 1 h after TCDD exposure [10], we believe that identification of primary *Ahr *responders is only possible by including early time points of exposure in gene expression studies.

In this work, we investigate the hypothesis of whether time-resolved transcriptional signatures of genes that are primary *Ahr *targets differ from the profiles observed for genes responding to the toxic metabolite BPDE. We demonstrate that machine learning can be used for identifying these characteristic signatures and for subsequently classifying genes as to whether they are primary *Ahr*-dependent targets or indirectly affected (BPDE-dependent) genes. This general strategy of using time course gene expression data to predict transcriptional regulatory roles has been previously explored [11-14], although primarily in lower organisms such as bacteria and yeast.

We expect that because such learning methods are less encumbered by methodological assumptions (compared to traditional statistical comparisons), they are more able to find subtle but meaningful patterns in the data. For example, an important assumption of previous attempts to cluster *Ahr*-centric expression data [3,15-17] is that co-regulated genes should also be co-expressed. Hence, clustering of genes based on expression patterns should identify sets of genes subject to the same regulatory program. However, in time courses such co-expression may only be present during certain phases. In the case of *Ahr *we expect co-expression during early time points, whereas expression may diverge later when the influence of *Ahr *diminishes. The analysis presented here anticipates and effectively deals with this scenario.

Here we employ machine learning techniques coupled to a straightforward yet robust experimental design in order to more clearly define genes that are under the direct transcriptional control of *Ahr*. This is accomplished by training a Random Forest [18] (RF) classifier to learn the difference between genes responding to B[a]P exposure and side effects caused by the B[a]P metabolite BPDE. The trained classifier is then applied to all genes found to be significantly differentially expressed as a result of B[a]P exposure, and their roles as primary responders or side effects are predicted. In addition, the patterns learned by the classifier are used as a basis for performing weighted clustering. These clusters facilitate a better understanding of the functional relatedness of the perturbed genes. Finally, we support predictions with our own experimental follow-up, as well as with data from independent studies.

## Results

### Extensive transcriptional response

The transcriptional response due to *Ahr *activation by 50 nM and 5 *μ*M B[a]P was investigated in murine hepatoma cells (Hepa1c1c7). Exposure effects were examined in time-course data for 2, 4, 12 and 24 h after treatment, together with corresponding vehicle (DMSO) controls.

A total of 2,338 genes were perturbed significantly (*FDR <*0.05) by exposure to B[a]P and had at least a 2-fold change (with respect to DMSO-exposed cells) at some time point over the course of the experiment. Compared to previous studies of *Ahr*-mediated temporal gene expression, this represents a very substantial transcriptional response (see Additional File 1, Table S1). These genes were highly enriched for a host of biological processes (summarized in Additional File 1, Table S2), including mRNA transport, control of the cell cycle, apoptosis, and development.

### Prediction of primary vs. side effects

**Figure 2:**
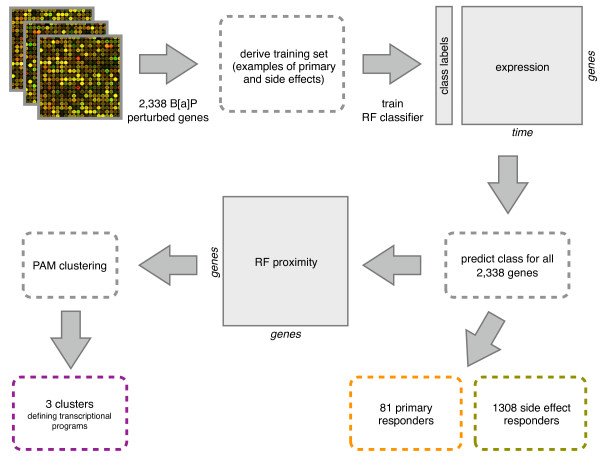
**Framework for predicting Ahr targets**. Framework for predicting *Ahr *primary responders and side effects using gene expression time course data.

The overall analytical framework used here is summarized in Figure 2. Using a matrix of time-resolved gene expression values as predictors (interpolated as described in methods), we trained a Random Forest classifier in a two-class scenario (*Ahr *primary and side effect). Training labels were assigned based on the significant perturbation of a gene in conditions that suggest being either a primary *Ahr *responder or responsive to the presence of BPDE (side effect). This yielded 28 genes as primary responders and 559 genes as side effects (Additional File 1, Figure S2), before filtering for outliers. The final classifier had an estimated misclassification rate of 7%. Performance of the classifier on out-of-bag (OOB) data is depicted as a receiver operating characteristic (ROC) curve in Additional File 1, Figure S3, panel A.

We then used this trained classifier to predict on all of the 2,338 differentially expressed genes. The predictions have varying degrees of confidence, indicated by the proportion of votes cast for the predicted class. To establish a threshold above which we could be confident that the classifier was predictive, we permuted the original training labels randomly, trained a Random Forest with these labels, and predicted on all 2,338 genes. In general we found that in this "null" scenario, the Random Forest did not predict with a proportion of votes greater than 0.8. Therefore, we consider a class prediction with a proportion of votes greater than 0.8 to be a reliable prediction (Additional File 1, Figure S3, panel B). After filtering, 81 genes were predicted as primary responders to *Ahr *(Table 1), 1,308 genes were predicted as side effects, and 949 genes could not be reliably classified (see Additional File 2).

**Table 1:** List of predicted primary targets of Ahr.

MGI ID	cluster votes	target votes	train/test	MGI ID	cluster votes	target votes	train/test
Hspa4l	1.00	0.98	test	Ccng2	0.98	0.9	test
2410066E13Rik	1.00	0.98	test	Fam198b	0.98	0.9	test
Arl6ip5	1.00	0.98	test	Ddit4	0.98	0.9	test
Plscr2	1.00	0.98	test	Ubl3	0.98	0.87	test
Mpp2	1.00	0.98	training	Nqo1	0.98	0.87	test
Tiparp	1.00	0.98	training	Trp53inp1	0.98	0.87	test
Sdpr	1.00	0.98	test	Cyp1a1	0.98	0.86	test
Ndrg1	1.00	0.97	test	Abca6	0.98	0.86	test
Nrn1	1.00	0.97	test	Hmox1	0.98	0.83	test
Cyp2s1	1.00	0.97	test	Aldh4a1	0.98	0.81	test
Tnfaip2	1.00	0.97	test	Npffr1	0.97	0.91	test
Cpox	1.00	0.97	training	**Btg2**	0.97	0.89	test
Osbpl2	1.00	0.97	test	**Nr3c1**	0.97	0.87	test
Rbks	1.00	0.96	test	Gm10122	0.97	0.87	test
**Ppard**	1.00	0.96	training	Snx30	0.96	0.96	training
Tbc1d16	1.00	0.95	test	Cdkn1b	0.96	0.92	test
Arrdc3	1.00	0.95	training	Slc26a2	0.96	0.88	test
**Lpin1**	1.00	0.95	test	Plk2	0.96	0.85	test
**Id2**	1.00	0.94	test	**Zscan29**	0.96	0.83	test
Xdh	1.00	0.94	test	Zfp608	0.95	0.92	training
Gramd3	1.00	0.94	test	Nrg1	0.95	0.91	test
Serpine1	1.00	0.93	test	Abcd2	0.95	0.8	test
Pfkfb3	0.99	0.98	training	**Klf9**	0.94	0.94	test
Jub	0.99	0.97	test	Dusp1	0.94	0.92	training
Ddx58	0.99	0.97	training	Tnfaip8	0.94	0.88	test
Zfp418	0.99	0.95	test	9330175E14Rik	0.94	0.82	test
Sgk1	0.99	0.94	test	Lrrc30	0.93	0.89	test
**Jun**	0.99	0.93	test	Eda2r	0.93	0.85	test
Cdkn1a	0.99	0.92	test	Bmf	0.92	0.93	test
Abcc4	0.99	0.91	test	Rnf39	0.91	0.92	training
Slc6a9	0.99	0.91	test	St6gal1	0.9	0.94	training
Adh7	0.99	0.90	test	Zfp36l1	0.89	0.83	test
Usp18	0.99	0.90	test	**Nr1d1**	0.86	0.91	training
Npc1	0.99	0.88	test	Irs2	0.86	0.91	test
Casp3	0.99	0.87	test	**Ets2**	0.84	0.86	training
Aldh3a1	0.99	0.86	test	**Nfe2l2**	0.78	0.86	test
Slc35d1	0.99	0.85	test	**Irf1**	0.76	0.91	training
Cyp1b1	0.98	0.97	test	Cib2	0.71	0.84	test
Intu	0.98	0.95	training	S1pr1	0.7	0.89	training
Pitpnc1	0.98	0.95	training	Traf5	0.59	0.89	training
Sesn2	0.98	0.92	training				

List of predicted primary targets of *Ahr *transcriptional regulation. Confidence scores of both membership in cluster 3 (cluster votes) and as a primary *Ahr *target (target votes) are given. Known transcriptional regulators (i.e. annotated with relevant GO terms) are bolded. Additionally, assignment to either the training or test set is indicated for each gene.

### Characterization of transcriptional response programs

**Figure 3:**
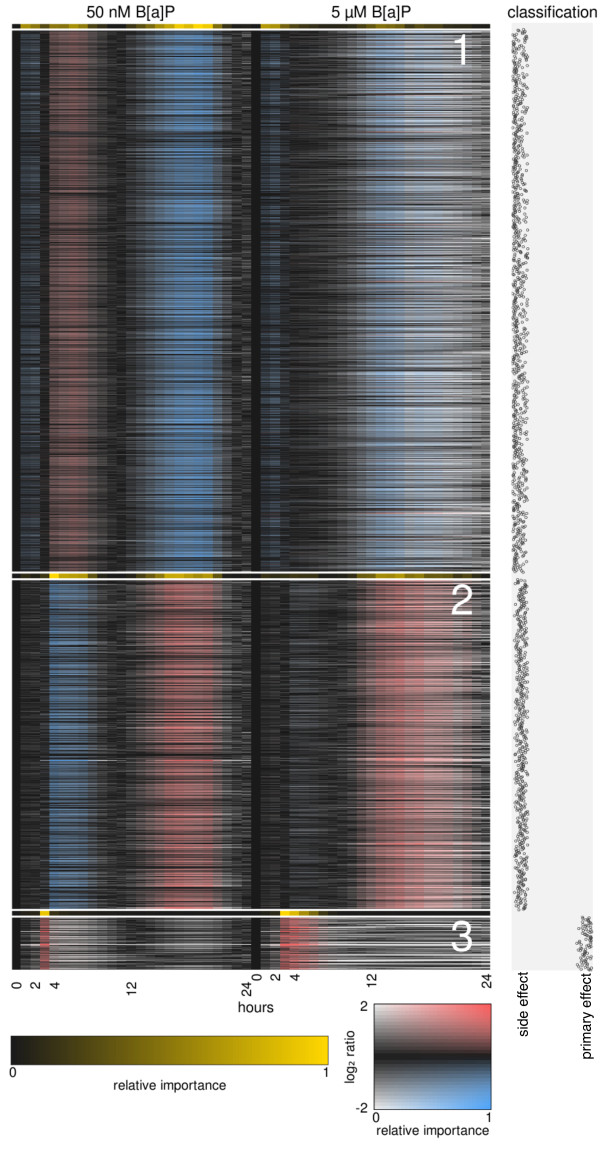
**Clustering with the RF proximity measure**. PAM clustering was performed with a supervised, weighted distance measure, derived during the classification of *Ahr *primary responders and side effects. Three distinct programs were found, depicted here as clusters (1-3). Color saturation indicates the importance of the time points for the identity of the cluster. To further emphasize these important time points, this same information is shown again for each cluster (black to yellow scale). The classification of each gene is shown as the proportion of RF votes.

To characterize the expression patterns that underlie the classifier's decision rules, we used the RF proximity measure as an input to PAM (partitioning around medoids) clustering - this combination is a form of weighted clustering. This yielded three coherent clusters, depicted in Figure 3. Clusters 1 and 2 are comprised of genes predicted to be side effects of *Ahr *activation by B[a]P, while cluster 3 contains genes predicted to be primary responders to *Ahr*. Clusters 1 and 2 are characterized by undulating expression profiles in the low (50 nM) B[a]P exposure, with the mean behavior of each cluster strongly anticorrelated to the other. The high (5 *μ*M) B[a]P exposure shows less cohesive expression patterns, but with the same general trend of anitcorrelation between clusters 1 and 2. In both cases, time points in the 50 nM B[a]P series are more important for the identity of the clusters than time points in the 5 *μ*M B[a]P series. Cluster 3 is characterized by punctuated expression induction at 3 hours in the 50 nM B[a]P time series, and a slightly extended phase of induction in the 5 *μ*M B[a]P time series. Other time points are unimportant for the cluster's identity; indeed, the expression of these genes is fairly divergent outside of the common phase of induction. Although cluster 3's "identity phase" is generally between 3-4 hours after exposure, where all genes in the cluster show elevated expression, several genes (such as *Cyp1a1 *and *Tiparp*) in the cluster are highly expressed well before this window.

**Figure 4:**
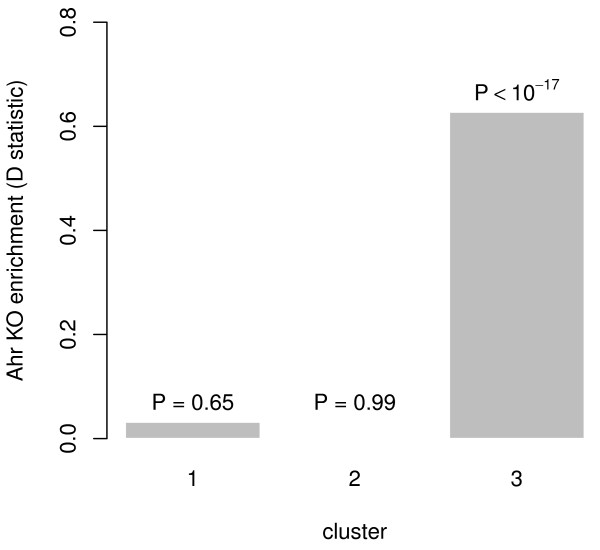
**Enrichment of each cluster for Ahr mutant-perturbed genes**. Using data from previous *Ahr *mutant studies [7,19], we assessed whether each cluster was enriched (relative to the other clusters) for genes perturbed by an *Ahr *mutation. Only genes not used in the training of the classifier were used in the calculation of enrichment. Cluster 3 was highly enriched for perturbed genes, suggesting that it is enriched for *Ahr *targets.

Using the Kolmogorov-Smirnov (KS) test, we evaluated the clusters for enrichment of genes perturbed by an *Ahr *mutation (Figure 4). By using data from previous studies [7,19], we performed a 2-way ANOVA and took *P *values from the genotype*ligand interaction; these *P *values were used as indicators of genes under the direct influence of *Ahr*. Genes belonging to the training set were excluded when calculating the enrichment. Cluster 3 was the only cluster to show enrichment for genes perturbed by an *Ahr *mutation. This result further supports the assertion that cluster 3 contains true *Ahr *primary responders, and that the classifier is predictive in practice. We similarly checked the three clusters for overrepresentation of known XRE motifs, using UCSC 5 kb upstream promoter sequences and motifs from TRANSFAC (release 2009.3). We found only borderline (*P *= 0.056) enrichment of an XRE motif among genes in cluster 3 and no enrichment in the other clusters. The lack of significant enrichment among the predicted primary *Ahr *responders suggests that our knowledge of the sequence-level requirements for functional binding of *Ahr *is currently far from complete.

### Experimental confirmation of Ahr dependency

Two independent experimental approaches were chosen to confirm *Ahr*-dependency for a subset of representative genes: direct comparison of the transcriptional response of *Ahr*-expressing Hepa1c1c7 and mutant tao BpRc1 cells deficient in endogenous *Ahr*, as well as confirmation of binding of *Ahr *in the corresponding promoter regions by chromatin immunoprecipitation (ChIP).

B[a]P is likely to induce side effects caused by B[a]P metabolites independent of direct *Ahr *activation, therefore we included TCDD - a non-metabolized *Ahr *ligand - in our confirmation experiments. Differential expression of *Tiparp*, *Tnfaip2*, *Cdkn1a*, *Cdkn1b*, *Cyp2s1*, *Nfe2l2*, *Mpp2*, and *Klf9 *after treatment with B[a]P or TCDD at different concentrations was investigated by quantitative real-time PCR (qPCR). After B[a]P and TCDD exposure, the expression of all genes was induced as soon as 1 h after the start of treatment in Hepa1c1c7 cells, while there was no significant induction compared to vehicle control samples detectable in tao BpRc1 cells up to four hours after exposure (Additional File 1, Figure S4). To complement these experiments, the effect of BPDE treatment on the predicted primary *Ahr *targets was investigated. After 2 h of exposure to 5 *μ*M BPDE, a time point for which pronounced induction with B[a]P was observed, no significant upregulation of these genes by BPDE exposure was found (see Additional File 1, Figure S9).

Enrichment of *Ahr *binding in the promoter region of all chosen genes could be confirmed by ChIP, with fold changes (compared to vehicle control samples) ranging from 7.2-152.2 (Additional File 1, Figure S5).

To investigate the impact of *Ahr *itself on the diverged pattern of the low and high B[a]P concentration in the later time course (Additional File 1, Figure S6) we studied *Ahr *nuclear translocation over time. GFP-*Ahr *expressing cells (genetic background: tao BpRc1) were exposed to either 50 nM or 5 *μ*M of B[a]P for up to 24 h. Nuclear translocation was determined as the ratio of nuclear to cytoplasmic fluorescence. A prolonged nuclear translocation of the receptor was detectable for the high concentration, whereas after 24 h of exposure to 50 nM B[a]P the distribution of single cell ratios approximated the control distribution (Additional File 1, Figure S7). This finding suggests a continuous transcriptional activation by *Ahr *with the higher concentration of B[a]P corresponding to a prolonged induction of *Ahr *target genes as, seen in cluster 3 for 5 *μ*M B[a]P.

### Confirmation of BPDE dependency

To confirm that the differential expression observed for genes of either cluster 1 or 2 is indeed dependent on BPDE, we used the previously mentioned *in vitro *set-up (Hepa1c1c7 vs. tao Bprc1 cells). The relevant BPDE concentration was determined by comparing the effect of 5 *μ*M B[a]P on Hepa1c1c7 cell proliferation to that of different BPDE concentrations in the same cell line. While BPDE concentrations up to 1 *μ*M had only a marginal effect on proliferation, 5 *μ*M BPDE induced an effect very similar to that seen with 5 *μ*M B[a]P (Additional File 1, Figure S1). Therefore, subsequent qPCR experiments for representative genes were performed with 5 *μ*M B[a]P or BPDE respectively. The chosen genes showed transcriptional responses in *Ahr*-deficient cells only when exposed to BPDE itself. In Hepa1c1c7 cells B[a]P treatment induced effects similar to BPDE, however with a pronounced time lag (Additional File 1, Figure S8).

## Discussion

Exposing cells to xenobiotic compounds like drugs or environmental pollutants often induces a complex transcriptional response, made up of both specific and unspecific regulatory mechanisms. Distinguishing the transcriptional profiles associated with the primary target effect from those acting in parallel is essential for understanding possible side effects of such chemicals.

As an example of such a case, we investigated *Ahr*, one of the most prominent ligand-activated TFs involved in xenobiotic-induced signaling. The cellular response to *Ahr *activation can be seen as a mixture of a primary response and side effects. The side effects are due in part to stress caused by the formation of active metabolites of the *Ahr *ligand, while the primary response is related to *Ahr *binding to gene regulatory sequences. A subsequent transcriptional cascade downstream of *Ahr *might be activated by other TFs, which are themselves regulatory targets of *Ahr *(Figure 1).

We have employed a time-course design involving early and late time points to capture both primary and downstream effects. These effects are separated on the time axis, but it is not obvious *a priori *where to draw the line, i.e. up to which time point expression changes reflect primary responses. The use of machine learning allowed us to identify the relevant time points in a data-driven way. In addition to weighting time points with respect to their relevance for distinguishing *Ahr *target genes, this analysis also identifies specific expression patterns that are characteristic of primary *Ahr *targets.

### Ahr target genes

Previously well-described members of the *Ahr *gene battery like *Cyp1a1*, *Nqo1 Cyp2s1*, *Aldh3a1*, *Aldh4a1 *and *Cyp1b1 *[1,20,21] were predicted as primary responders to *Ahr*. In addition to this qualitative confirmation of the effectiveness of our computational approach, we could demonstrate *Ahr *dependency experimentally by chromatin immunoprecipitation (ChIP) and qPCR. Further, we found the set of predicted targets to be enriched for genes that showed significant *Ahr *genotype*ligand interaction (i.e. 2-way ANOVA) effects based on previously published data [7,19] (Figure 4).

B[a]P is likely to induce side effects caused by B[a]P metabolites independent of direct *Ahr *activation, therefore we included TCDD - a non-metabolized *Ahr *ligand - in our confirmation experiments. All of the genes chosen for the qPCR verification confirmed the predicted *Ahr*-dependency (Additional File 1, Figure S4).

We performed a GO enrichment analysis for a functional evaluation of the predicted target genes. The regulated genes in cluster 3 were enriched for 15 different biological functions including terms related to cell cycle control and proliferation. This influence on the cell cycle is also manifested on the protein level, as we were able to show in a previous study [22]. Experimental confirmation of two of these genes, the cyclin-dependent kinase inhibitors *Cdkn1a *and *Cdkn1b*, showed an exclusive induction in wild type cells, together with an enrichment for *Ahr *binding at the corresponding promoters. Another gene known to be involved in cell cycle regulation, but less well-defined, is the palmitoylated membrane protein 2 (*Mpp2*). *Mpp2 *was also strongly induced by TCDD and B[a]P in *Ahr*-expressing cells, while no differential expression was elicited in the mutant tao BpRc1 cells. A more indirect effect on cell cycle regulation originates from the TNF alpha activated signaling cascade. Five genes (*Tnfaip2*, *Tnfaip8*, *Traf5*, *Casp3*, *Ddx58*) involved in this pathway were predicted to be primary responders to *Ahr*.

*Tnfaip2 *and *Casp3 *were investigated in our independent experimental confirmation. For both genes induction of expression was only detectable in Hepa1c1c7 cells, while the *Ahr*-deficient counterparts showed no significant differential regulation. Actual binding of *Ahr *to the promoter sites could be confirmed by ChIP. Primary regulation by *Ahr *of the important regulators of the cell cycle *Cdkn1a*, *Cdkn1b *as well as *Mpp2 *together with targeting of the TNF alpha signaling pathway emphasizes the impact of *Ahr *on endogenous cellular functions outside of xenobiotic metabolism. Further, these findings suggest that the observed reduction in proliferation after exposure to B[a]P is not only a response to DNA damage, but is also, at least in part, a direct consequence of *Ahr *activation.

The early time points proved vital in distinguishing *Ahr *targets from genes induced as side effects (Figure 3), emphasizing the importance of planning experiments such that the immediate effects are captured. Although perturbation at early time points determined the *Ahr *primary response for both B[a]P concentrations, the consistency of expression between the concentrations diverged later in the time course (Additional File 1, Figure S6). We investigated if indeed *Ahr *itself might be important for this difference. Comparing the translocation behavior of *Ahr *we could show a persistent nuclear localization of *Ahr *for high B[a]P concentrations for up to 24h of exposure, while for low concentrations of B[a]P cells showed fewer and less pronounced translocation events. Obviously many mechanisms might be responsible for the concentration-dependent differences in the transcriptional pattern, like the balance between mRNA production and decay. Nevertheless, persistent *Ahr *translocation suggests persistent mRNA production, thereby shifting this balance.

### An Ahr transcriptional cascade

Twelve of the genes in cluster 3 (i.e. the *Ahr *target cluster) are known transcriptional regulators. These regulators could constitute a transcriptional cascade that begins with the activation of *Ahr*.

In a recent study, Dere et al. [23] integrated ChIP-chip and transcriptional data of murine liver tissue after TCDD exposure. Interestingly, over 70% of the genes we predicted by our approach as primary *Ahr *responders were also identified in their study to be located in regions of *Ahr *enriched binding. More importantly, eleven out of the twelve transcriptional regulators identified by our method were also found in such *Ahr *enriched binding regions. This not only underlines the quality of our Random Forest classifier, but suggests a more general transcriptional network initiated by *Ahr*, independent of the activating ligand.

*Ahr *has been connected to hormone-induced signaling as was reinforced by our GO enrichment analysis that identified "regulation of hormone levels" as one of the biological functions. Crosstalk with the estrogen receptor has been studied extensively [24-26] and glucocorticoid receptor (GR)/*Ahr *crosstalk has also been suggested [27,28]. Our classifier predicted the glucocorticoid receptor (*Nr3c1*) itself as an *Ahr *target together with *Sgk1*, a GR-regulated kinase. In addition, the TF *Klf9*, known to be induced by GR and involved in adipogenesis, was predicted to be a direct *Ahr *target. Besides *Klf9*, further *Ahr *targets were predicted with an involvement in lipid synthesis and lipid transport, i.e. the transcriptional regulators *Ppard *and *Lpin *play a role in mammary lipid synthesis, and *Npc1*, *Osbpl2*, and *Pitpnc1 *are involved in lipid transport. The role of GR in lipid homeostasis and metabolism is well-established [29-31]. From our analysis we can deduce a possible *Ahr*-activated network of genes directly influencing lipid status and its regulation by the glucocorticoid receptor.

The interaction of *Ahr *with another TF *Nfe2l2 *(aka *Nrf2*) might also have an influence on lipid status, specifically on adipogenesis [32]. A bidirectional regulation of these two pathways has been described previously [33]. Both TFs have been shown to bind in the other's promoter region, thereby directly influencing transcription [32,34]. Therefore, the prediction of *Nfe2l2 *being an *Ahr *target is very well corroborated by previous studies and was indeed verified by our experimental follow-up. In addition, a recently described interaction of *Nfe2l2 *and *Ahr *confirms one other predicted *Ahr *target gene: *Abcc4*. Xu et al. showed that this multidrug resistant protein is directly activated by *Ahr *and *Nfe2l2 *in liver [35].

In our analysis we were only able to reliably classify 1,389 of the 2,338 regulated genes as either primary *Ahr *targets or as genes responding to BPDE stress. We found that the unclassified genes were enriched (*P *= 0.019) for genes perturbed by an *Ahr *mutation. A possible explanation for this enrichment is that there are genes that are downstream targets of *Ahr *(e.g. via the other transcriptional regulators that are primary responders to *Ahr*; see Figure 1, panel C) among this set. Since the classifier was not trained on such examples of downstream *Ahr *targets, we expect that it would not reliably classify these genes.

### Side effects

Genes in clusters 1 and 2 are predicted to be perturbed not as a result of *Ahr *regulation, but by the presence of the metabolite BPDE. This genotoxic metabolite of B[a]P is known to cause DNA damage by DNA-adduct formation [36,37]. DNA repair processes are initiated, followed by re-initiation of DNA replication (one of the eleven GO categories enriched in cluster 1). Further, many MAP kinases were differentially regulated, and all of them are members of clusters 1 or 2. The idea that MAP kinases are *Ahr*-independent is supported by Tan et al. [38], who could show that *Ahr *ligands could activate MAPKs independent of *Ahr*.

To further support the predictions of our classifier, we selected some representative genes from clusters 1 and 2 (*Agfg1*, *Anapc1*, *Nfkb*, and *Parp1*) and measured their expression in response to exposure to B[a]P or BPDE in wild type (Hepa1c1c7) or mutant (tao BpRc1) cells (Additional File 1, Figure S8). These experiments demonstrate that BPDE causes differential expression with and without *Ahr*, while B[a]P only perturbs expression in the presence of *Ahr*, i.e. when metabolism of B[a]P to BPDE is made more efficient by a functional *Ahr *pathway. These results demonstrate, as predicted, that these genes are affected by the presence of BPDE and are not a primary response regulated by *Ahr*.

### Utility of weighted clustering

One unique and desirable aspect of the type of learning approach applied here is a side effect of the learning process - the proximity measure. The RF proximity is a type of similarity measure between subjects (in this case genes), based on how often two genes take the same path down the decision trees of the forest. It is in effect a weighted similarity measure because only time points that are useful in the learning process are used in the calculation of the proximity. This is in contrast to the widely used Euclidean distance or Pearson correlation, in which all features make an equal contribution.

A weighted (dis)similarity measure is advantageous in clustering gene expression time series, especially in complex transcriptional responses of higher eukaryotes, as presented in this work. Additional systems are present in higher eukaryotes that influence the synthesis, stabilization, and degradation of mRNA. These additional systems make it less likely that functionally related genes share precisely the same characteristic expression profile over time. For instance, functionally related genes, induced by a common TF, may share similar expression patterns shortly after induction, but may then diverge as other factors come into play, such as microRNAs. A supervised, weighted metric such as RF proximity de-emphasizes the diverging time points while emphasizing the common phase of induction, resulting in the grouping of the functionally related genes. Conversely, such expression profiles are unlikely to fall into the same cluster when using e.g. the Euclidean distance, and could be a contributing factor to the mixed success of past attempts [3,15-17] to cluster *Ahr*-induced gene expression time courses in a way that is biologically interpretable.

One technique that is frequently used to address problems such as those described here is biclustering [39-43]. Briefly, biclustering is a strategy that seeks to cluster in two dimensions simultaneously, e.g. genes and time points. The goal is to find genes that show similar expression in some (though not necessarily all) conditions. There are many algorithms and heuristics that implement biclustering. Strengths and weaknesses of the approach vary by implementation, but in general most biclustering methods are unsupervised and are non-deterministic. Without alleviating assumptions it can become a computationally intractable problem. It can be difficult to judge the quality of the resulting clusters, and clusters are often redundant. In the work presented here, clustering with the RF proximity presented fewer potential pitfalls compared to biclustering, since we had a means of performing supervised learning and the RF proximity was obtained "for free" since it was part of the learning process. In addition, the clusters were non-redundant and judging their quality was fairly straightforward by using another Random Forest to predict the assigned cluster labels of the genes (as described in the methods section). In addition to the work presented here, clustering with an unsupervised RF proximity has been described in Shi and Horvath [44], and an example using multivariate response Random Forests to examine transcriptional programs in yeast can be found in Xiao and Segal [45]. We have found that PAM clustering with the RF proximity measure works well in scenarios where weighted clustering is desirable, and is an alternative to biclustering that is worth considering. However, one obvious limitation for any supervised method - including our use of RF here - is the need for a training set. In some situations a training set may be difficult or impossible to assemble - this is an important consideration when selecting a clustering method.

## Conclusion

We explored the time-resolved transcriptional response induced by exposure to the environmental pollutant B[a]P and mediated by the transcription factor *Ahr*. As with many microarray experiments involving cellular stress, we observed an immense degree of differential expression, which often complicates biological interpretation. However, by using machine learning approaches, we successfully teased apart the specific, receptor-driven transcriptional response from the more general toxic response. Genes predicted to be part of a primary receptor-driven response were validated by extensive experimental work, further supporting the predictive power of our classifier. In addition to the specific results that further characterize the *Ahr *regulatory battery, our work here offers a useful strategy for distinguishing receptor-dependent responses and side effects based on expression time courses.

## Methods

### Cell culture and sample preparation

Murine hepatoma cells, Hepa1c1c7 as well as the mutant tao BpRc1 cells (both LG Standards GmbH, Wesel, Germany), deficient in endogenous *Ahr*, were used for all experiments. Cells were cultured in phenol red-free DMEM supplemented with 7% FCS, 1% glutamine and 1% penicillin/streptomycin. *Ahr *translocation was investigated in a stable cell line based on tao BpRc1 cells expressing GFP-*Ahr *under tetracycline control. Cells were stimulated with different concentrations of benzo-[a]-pyrene (B[a]P; Sigma Aldrich, Steinheim, Germany), BPDE (Midwest Research Institute, NCI Chemical Repository, Kansas City, MO, USA) and TCDD (Sigma-Aldrich, Steinheim, Germany) dissolved in DMSO respectively.

### Microarrays

To investigate the differential kinetic behavior of the transcriptome after B[a]P exposure, and to identify the primary *Ahr *response, we used two different setups: (1) short term exposure, Hepa1c1c7 cells were treated with 50 nM B[a]P for 0, 1, 2, and 4 hours and (2) long term exposure, Hepa1c1c7 cells were treated with 50 nM or 5 *μ*M B[a]P for 2, 4, 12 and 24h. Corresponding time-matched vehicle controls were generated. All experiments were performed in triplicate. Cells were lysed in Trizol reagent (Invitrogen, Darmstadt, Germany) and RNA extracted using RNAeasy kits (Qiagen, Valencia, CA, USA). RNA was quantified and integrity verified on a Bioanalyzer (Agilent Technologies, Palo Alto, CA). Sample preparation for Affymetrix GeneChip Mouse Exon 1.0 ST arrays (Affymetrix, Santa Clara, CA, USA) was performed following the manufacturer's recommendations. Microarray data was deposited in the Gene Expression Omnibus (GEO) under the identifier GSE29188.

### Detection of differential expression

Microarrays were normalized using RMA and the University of Michigan custom CDF file (version 12.1.0) with mappings to Ensembl exon IDs. After normalization, but before proceeding with the analysis, we subtracted the (log_2_) DMSO expression values from the corresponding time point and batch of each of the B[a]P treatments. Exon expression values were then summarized to their corresponding Ensembl gene IDs, with the summarized gene expression value being the mean of its constituent exons. A 2-way ANOVA analysis was performed on each gene, with time and concentration as the factors. We then corrected for multiple testing by using the FDR. We considered only genes with an *FDR <*0.05 for any of the main effects or time*concentration interaction. In addition, we admitted genes with an *FDR <*0.05 from a simple t-test each B[a]P concentration (all time points pooled) vs. DMSO. Of these, we only considered genes that achieved 2-fold (or greater) differential expression at at least one time point. This left us with a total of 2,338 genes regulated in the long-term exposure (24 hour) data set. We interpolated the expression between the measured time points by averaging the simple linear interpolation with the spline interpolation. Since we have no measurement at time 0 hours, we assume equivalent expression with the DMSO samples, i.e. the expression ratio at time 0 hours is 0 on the log_2 _scale. The interpolation gave us a total of 25 values per gene, 1 value every hour from 0 to 24 hours. Whether or not the expression values were interpolated did not significantly affect the results of the classification and clustering, but we opted to use interpolated values to aid in visualization and interpretation.

### Classification with Random Forests

We used the R implementation of Random Forests [46] to perform the two-class classification (*Ahr *primary response vs. side effects), using the time course expression measurements of significantly regulated genes as predictors. To derive training labels (Additional File 1, Figure S2), we used data available from two BPDE studies in human cell lines [47,48], combining the *P *values from the studies using Fisher's method. We labeled mouse orthologs of genes with BPDE-perturbed expression (*FDR <*0.05) as "secondary" since BPDE does not bind *Ahr*, but indicates affected genes further downstream of *Ahr*. We labeled genes as "primary *Ahr*" that showed differential expression (*FDR <*0.05) in an independent gene expression time course of cells exposed to 50 nM B[a]P from 0 to 4 hours, with the additional condition that they were not significantly regulated in the BPDE data (i.e. orthologs had *FDR >*0.05). These criteria led to 28 "*Ahr*-primary" labeled genes and 559 "side effect" labeled genes.

With this training set we ran RF with mtry set to 5, and ntree set to 5,000. We used the built-in outlier measure and removed genes in the 95*^th ^*percentile of outlier scores (resulting in 27 primary response and 530 side effect training cases), then re-ran RF, this time with 1,000 trees. In both cases, to avoid biased predictions (since there are far more "secondary" samples) we randomly sampled 20 genes from each class for the construction of each tree in the forest. The overall misclassification rate for the final forest was 7% (out of bag error estimate).

Predictions were made for all 2,338 differentially expressed genes, and genes with a proportion of class votes greater than 80% were retained for further analysis. This cutoff was chosen because when the training labels were permuted randomly and a RF trained, no prediction had a proportion of votes greater than 80%. Using these criteria, a total of 82 genes were predicted to be responding to *Ahr *directly, and 1,365 genes were predicted to be side effects (e.g. regulated through the presence of B[a]P metabolites). In addition to predictions, the RF proximity measure was calculated for all significant and confidently classified genes, yielding a 1,447 by 1,447 matrix.

### Clustering

The RF proximity matrix was used as a distance measure by the transformation $D=\sqrt{1-P}$, where *P *is the original proximity matrix and *D *is the distance matrix. This distance matrix was then used as the input for PAM clustering, available in the R cluster package. We tested a range of *k *values and found that specifying 3 clusters gave the best average silhouette.

To assess the degree of confidence in cluster assignment for each gene, an RF was fit to predict cluster label using the gene expression measurements. The proportion of votes for the correct cluster is an indication of how well a gene fits in the cluster. Genes that were given a lower proportion of votes for the correct class than expected under the null hypothesis (labels permuted randomly) were excluded. When including this additional filtering criterion, the final number of genes classified as primary responders was 81, with 1,308 genes as side effects. In addition, the importance measurements obtained in the construction of this RF give an indication of which time points and which concentrations are important parts of the cluster's identity.

GO enrichment was performed for each cluster (Additional File 1, Table S3) using the topGO package [49]. Enrichment of the clusters for genes perturbed by an *Ahr *mutation was performed using the Kolmogorov-Smirnov test, using *P *values derived from differential expression of genes from [7,19]. *P *values were calculated for each study separately, then combined using Fisher's method. Genes used to train the RF classifier were removed prior to calculation of enrichment, to ensure that the results reflected the actual predictive ability of the classifier.

### Cell proliferation

Long-term exposure studies in Hepa1c1c7 cells treated with B[a]P versus BPDE were performed using the xCELLigence System (Roche Diagnostics, Mannheim, Germany). This system measures electrical impedance across micro-electrodes integrated on the bottom of 96-well tissue culture E-plates (Roche Applied Science, Germany). Shifts in impedance are measured in real time, indicating changes in cell proliferation. Cells were monitored every 15 min for up to 24 h after treatment with 50 nM, 500 nM, 1 *μ*M, 2.5 *μ*M or 5 *μ*M of B[a]P or BPDE respectively. Each experiment was performed in triplicate.

### qPCR

In a separate experiment Hepa1c1c7 and tao BpRc1 cells were exposed to B[a]P (50 nM, 5 *μ*M), BPDE (50 nM, 5 *μ*M) and 1 nM TCDD for 0.5, 1, 2, and 4 h. mRNA was extracted and isolated using the MagNA Pure LC System (Roche Diagnostics GmbH, Mannheim, Germany). 50 ng of mRNA was reverse transcribed according to the protocol provided with the AMV reverse transcriptase (Promega, Madison, WI, USA). Resulting cDNA was diluted 1:5 and 4 *μ*l of template used in a 12 *μ*l PCR reaction. qPCRs were performed for the following example genes: *Tnfaip2*, *Tiparp*, *Cdkn1a*, *Cdkn1b Mpp2*, *Cyp2s1*, *Nfe2l2*, *Klf9*, *Lig3*, *Myst2*, *Axin2*, *Agfg1*, *Anapc1*, *Nfkb1*, *Parp1*, and the housekeeping genes 18S rRNA and *Gapdh *(primer sequences, Additional File 1, Table S1). All qPCR experiments were carried out on a LightCycler^®^480 system (Roche Diagnostics GmbH, Mannheim, Germany) with the following settings: touchdown amplification with an initial step of 96°C for 10 min; followed by the first cycle at 95°C for 10 sec. The annealing step started at 68°C for 20 sec (decrease of 0.5°C/cycle with a step delay of 1 cycle) and reaching the annealing temperature of 58°C for the last 25 cycles, followed by 72°C for 20 sec for extension. A total of 45 cycles were performed in each experiment.

### ChIP

Hepa1c1c7 cells were exposed to 50 nM B[a]P or DMSO as the vehicle control for 1 h. Subsequently, cells were exposed to 50 nM B[a]P or DMSO as the vehicle control for 1 h respectively. Subsequently cells were cross-linked for 10 min at 37°C in 1% formaldehyde followed by a quenching step for 10 min with 150 mM glycine. After cross-linking, chromatin DNA was sheared into 200-500 bp fragments by sonication using a Bioruptor^®^Next Gen (UCD-300, Diagenode SA, Liege, Belgium). Sonicated, soluble chromatin was immune-precipitated with 2.5 *μ*g of an anti-*Ahr *antibody (Enzolifesciences/Biomol, Lörrach, Germany) or anti-Pol II (Millipore, Billerica, MA, USA). Control IPs were performed using rabbit IgG (Millipore, Billerica, MA, USA) corresponding to our specific antibodies. DNA isolates from immunoprecipitates were used as templates for real-time quantitative PCR amplification using the primer pairs listed in Additional File 1, Table S2. All ChIP experiments were performed at least two times.

### Ahr translocation

Stably transfected tao BpRc1c cells, expressing a GFP-tagged *Ahr *under tetracycline control, were used to investigate the differences in translocation behavior for different concentrations of B[a]P. Cells were seeded in 96-well imaging plates (BD, Franklin Lakes, NJ, USA) and taken off tetracycline 24 h before exposure to allow for sufficient GFP-*Ahr *expression. Final B[a]P concentrations were 50 nM and 5 *μ*M respectively, including a corresponding DMSO control (0.05%). After treatment, cells were fixed using 3.7% formaldehyde, and the nuclei stained with Hoechst 33342 (Invitrogen, Darmstadt, Germany). Imaging was performed on a BD Pathway™Imager 855 in a non-confocal mode using a 20X U-Apo 340 objective (Olympus, NA 0.75). Images were binned 2 × 2 and montaged 2 × 2. Further analysis of fluorescence intensity was performed using the Attovision software (BD, Franklin Lakes, NJ, USA). After segmentation of the nucleus and the cytoplasm, the ratio of the nuclear and cytoplasmic fluorescence was calculated for each cell. Ratios were conflated in 0.01 intervals and relative frequencies determined. To allow for comparability the measurements were standardized so that the mean of the negative control equals 1. For the statistical analysis, more than 250 cells/treatment were considered.

## Authors' contributions

JM and ST wrote the manuscript. ST coordinated and performed experimental work. JM performed the computational work. FD and DM performed cell culture experiments in connection with the microarray data. Validation experiments were performed by SR (qPCR, ChIP) and C. Gräbsch (qPCR). JMa performed image analysis. KS, IL, MvB, SA, and AB conceived of the experimental design and functioned in an advisory capacity. All authors approved the final manuscript.

## Supplementary Material

Additional file 1 **supplementary information**. A PDF containing additional details on the experiments and analysis. Click here for file

Additional file 2 **cluster assignment and regulatory predictions for differentially expressed genes**. An XLS file containing the results of our RF classifier. Click here for file
