# Supplementary material for: Transcriptional signatures of regulatory and toxic responses to benzo-[a]-pyrene exposure
Source: BMC Genomics. 2011 Oct 13;12:502. doi: 10.1186/1471-2164-12-502 (PMC3215681; doi:10.1186/1471-2164-12-502)
Supplement: Additional file 1 — supplementary information. A PDF containing additional details on the experiments and analysis. [file 1471-2164-12-502-S1.PDF]

Transcriptional signatures of regulatory and toxic responses to chemical exposure (supplementary materials)

Jacob J. Michaelson, Saskia Trump, Susanne Rudzok, Carolin Gräbsch, Danielle J. Madureira, Franziska Dautel, Juliane Mai, Sabine Attinger, Kristin Schirmer, Martin von Bergen, Irina Lehmann, Andreas Beyer

Classification and clustering results Results of classification as an *Ahr* primary target or regulated by side effects, with cluster membership scores, are available in the supplementary data Excel file.

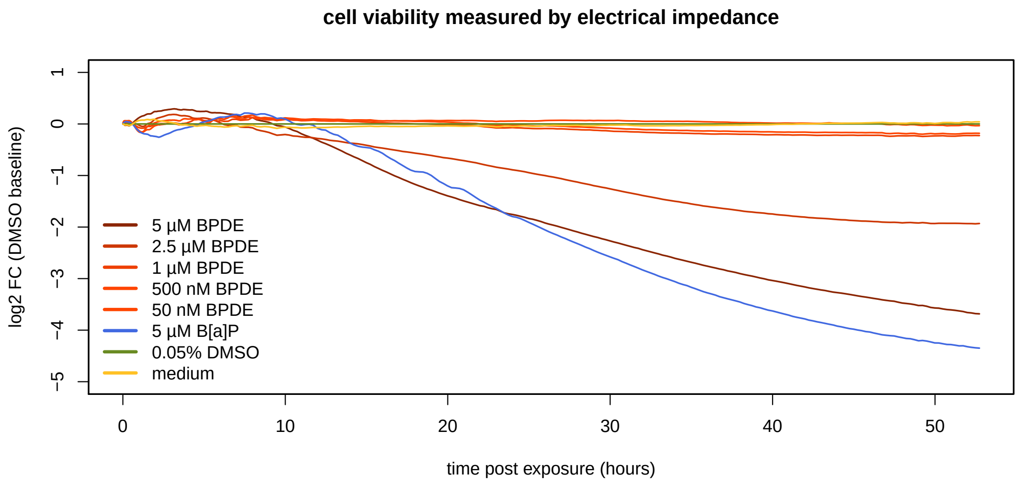

Figure S 1: Differences in proliferation after exposure to B[a]P or BPDE. Cell proliferation was measured in real-time via electrical impedance. Cells were treated with different concentrations of BPDE in comparison to 5 μM B[a]P. Proliferation curves were normalized to the value of DMSO treated cells at the corresponding time point.

study	organism	ligands	timepoints	# DE genes
Hockley, et al. (2007)	H. sapiens	TCDD, B[a]P, BPDE	6h, 24h	1,207
Hockley, et al.(2006)	H. sapiens	B[a]P, B[e]P	6h, 24h, 48h	202
Kim, et al. (2009)	H. sapiens	TCDD	1h, 2h, 4h, 8h, 12h, 24h, 48h	144
Dere, et al. (2006)	M. musculus	TCDD	1h, 2h, 4h, 8h, 12h, 24h, 48h	285
Frericks, et al. (2008)	M. musculus	TCDD	2h, 4h, 6h	201
Michaelson & Trump, et al.	M. musculus	B[a]P	2h, 4h, 12h, 24h	2,338

Table S 1: Overview of *Ahr*-centric time-resolved microarray studies. A brief description of the experimental factors is given, along with the total number of differentially expressed (DE) genes resulting from exposure. If multiple cell lines were tested, the maximum number of differentially expressed genes is reported.

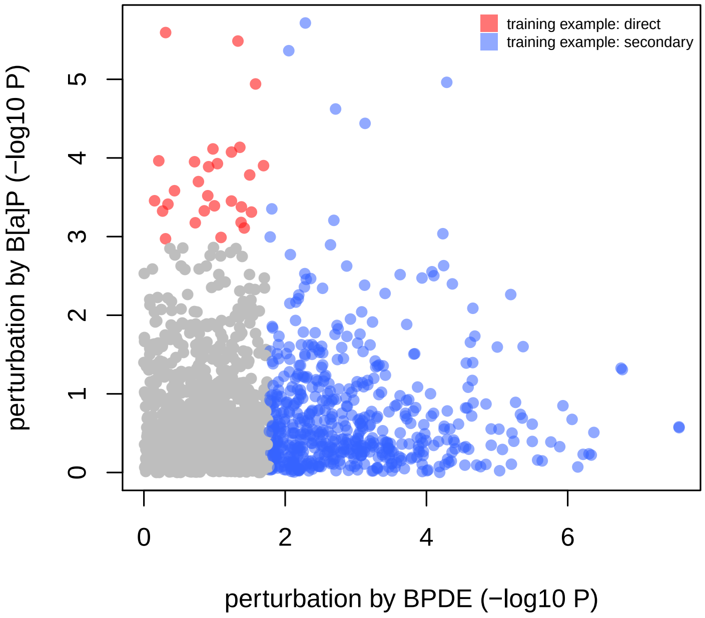

Figure S 2: Defining the training set. We used perturbation by BPDE ($FDR < 0.05$) as an evidence of a side effect, since BPDE does not activate *Ahr* but at the same time is a metabolite of B[a]P. Accordingly, perturbation by B[a]P ($FDR < 0.05$) but not by BPDE ($FDR > 0.05$) was taken as evidence for primary regulation by *Ahr*. A total of 1,663 genes of the 2,338 differentially expressed genes were examined as potential training examples, and 587 genes were then assigned to the training set.

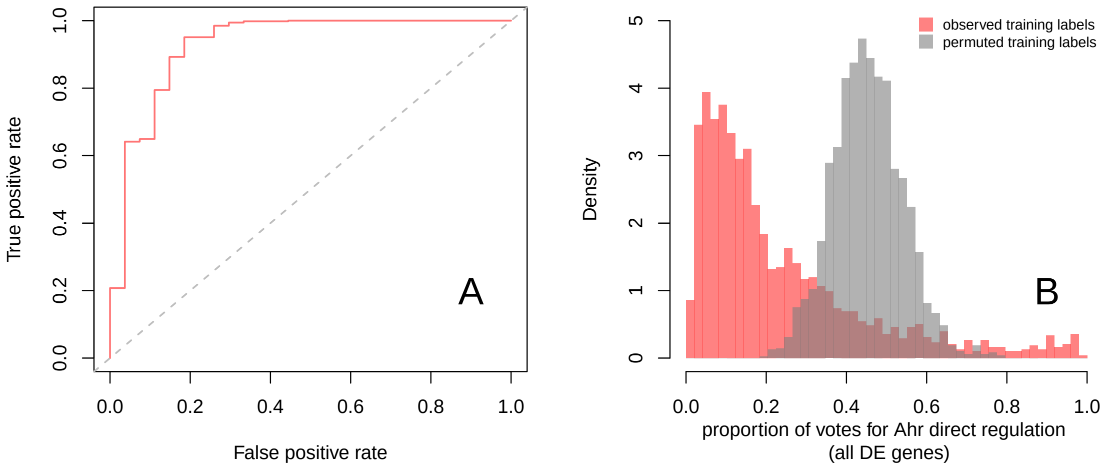

Figure S 3: Performance and confidence of predictions of the Random Forest classifier. Performance of the classifier predicting out-of-bag (OOB) data, depicted as a ROC curve (A). Confidence in class predictions can be expressed as the proportion of votes cast for the class (B). Here we show the actual proportions of votes of all differentially expressed (DE) genes (red), and the proportions when the training labels are permuted (grey). The classifier's predictions are more reliable for genes that have a proportion of votes outside of the overlapping region. Note that since this is a two-class scenario, a proportion close to 0 in this figure corresponds to a high proportion of votes for the gene being perturbed as a secondary effect.

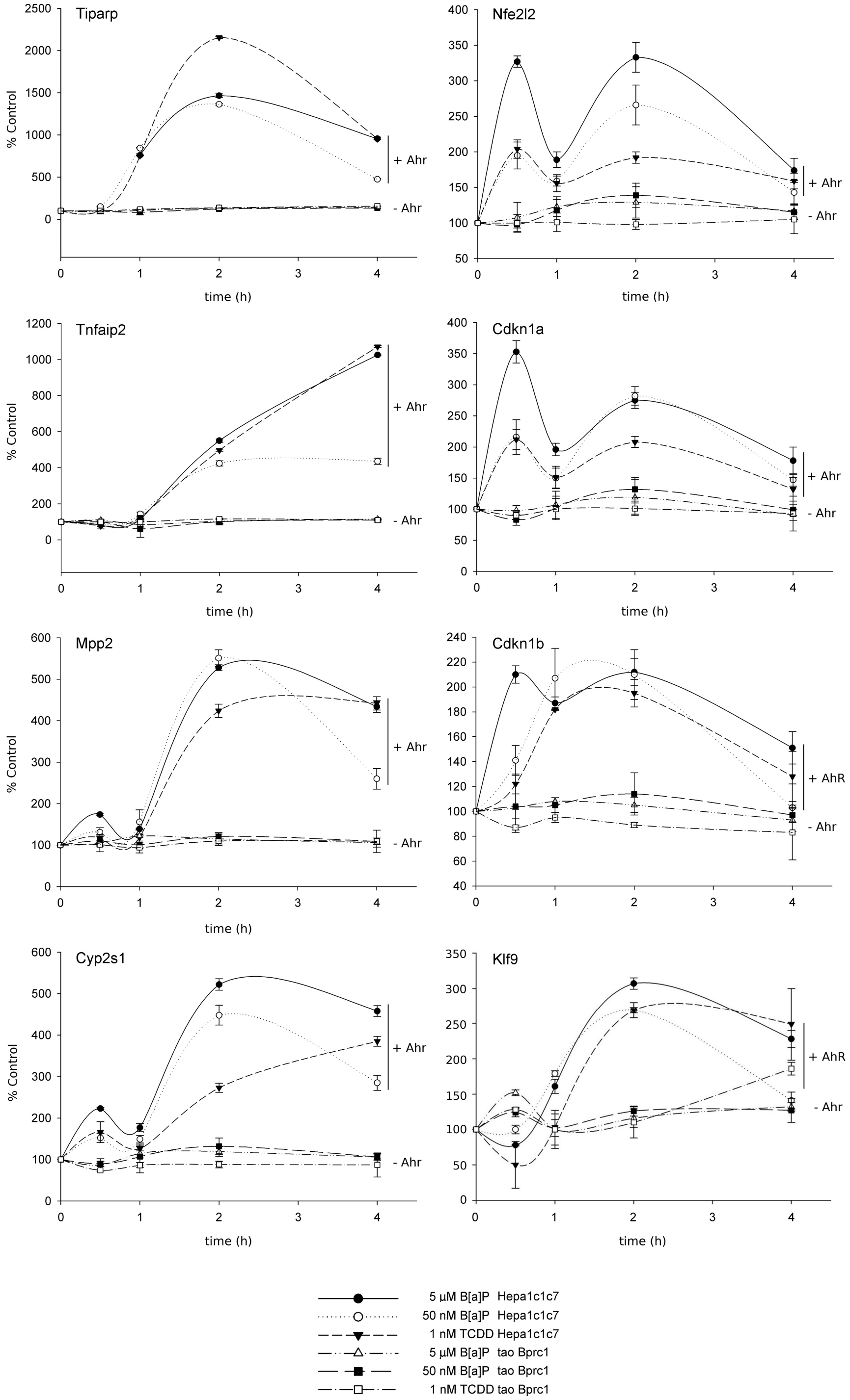

Figure S 4: Confirmation of predicted targets of *Ahr*. A subset of predicted primary *Ahr* target genes was confirmed by qPCR. Hepa1c1c7 (+*Ahr*) and tao BpRc1 (-*Ahr*) were exposed to two different concentrations of B[a]P or TCDD respectively. Significant differential expression of *Tiparp*, *Tnfaip2*, *Mpp2*, *Cyp2s1*, *Nfe2l2*, *Cdkn1a*, *Cdkn1b*, and *Klf9* was detected exclusively in *Ahr*-expressing cells (mean ± standard deviation are shown).

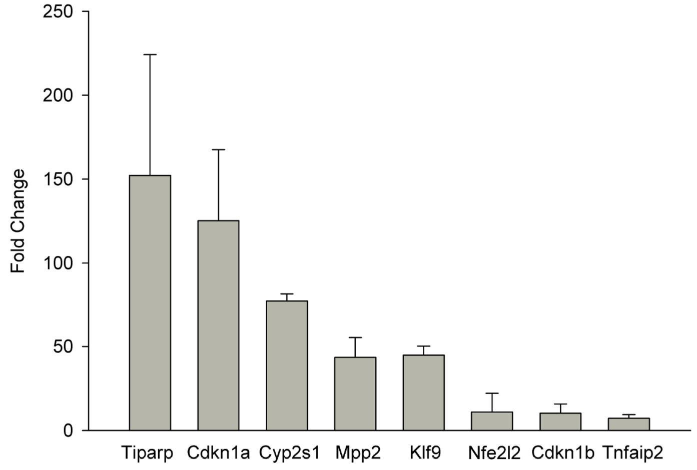

Figure S 5: Binding of *Ahr* after exposure to B[a]P to promoter sequences of selected predicted primary targets, assayed by ChIP. Fold change is relative to vehicle control (mean ± standard deviation are shown).

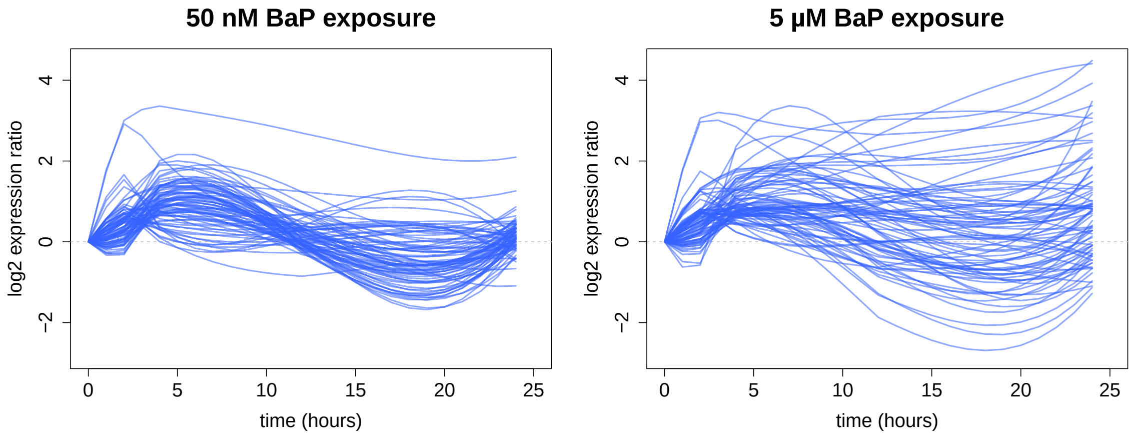

Figure S 6: Concentration-dependent differences in the transcriptional response of genes predicted to be *Ahr* primary targets.

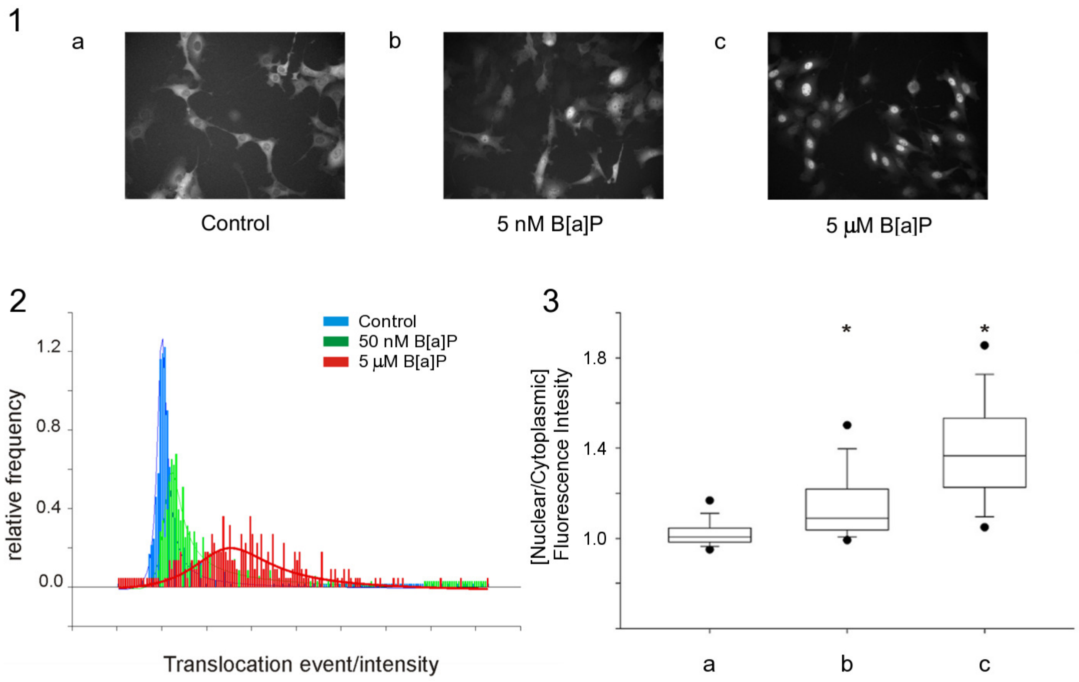

Figure S 7: Prolonged *Ahr* translocation with high concentrations of B[a]P. Cells were exposed to (a) DMSO, (b) 50 nM or (c) 5 μM of B[a]P for 24h and GFP-*Ahr* translocation was investigated by fluorescence microscopy (1). After segmentation of the nucleus and the cytoplasm the ratio of the nuclear and cytoplasmic fluorescence was calculated for each cell. Ratios were conflated in 0.01 intervals and relative frequencies determined (2), and boxplots show the distributions of these values (*$P < 0.001$, one-way ANOVA on ranks, followed by Dunn's method) (3).

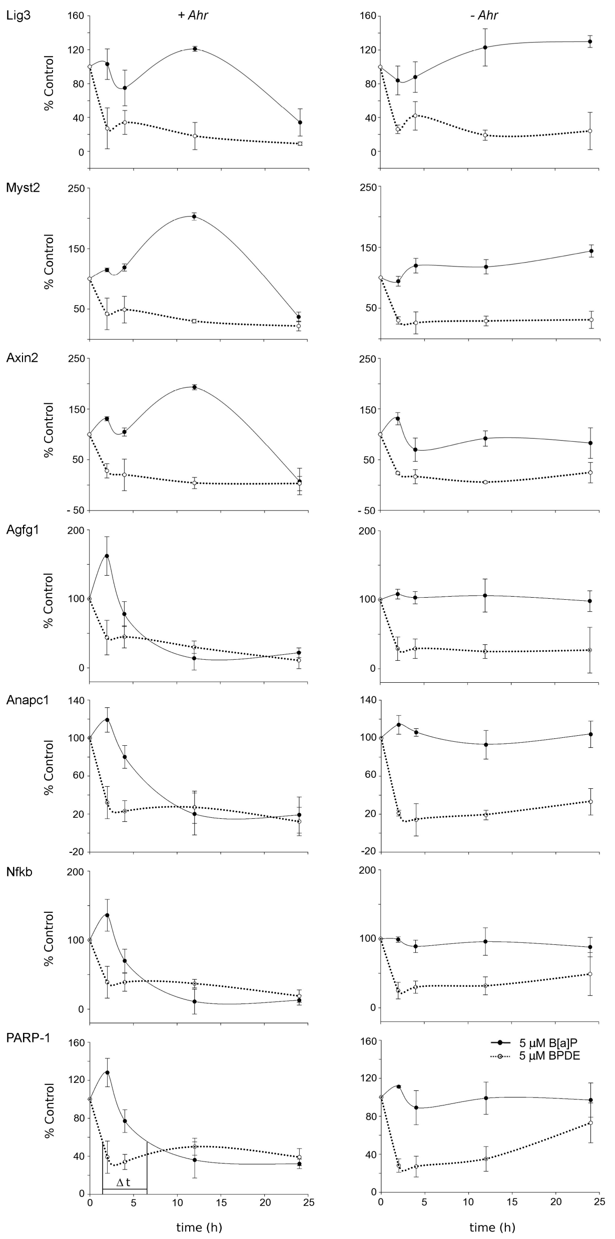

Figure S 8: Confirmation of predicted BPDE-perturbed genes. qPCRs for *Lig3*, *Myst2*, *Axin2*, *Agfg1*, *Anapc1*, *Nfkb*, and *Parp1*were performed in *Ahr* expressing (Hepa1c1c7, +*Ahr*) and in *Ahr* deficient cells (tao BpRc1, -*Ahr*). Cells were exposed to B[a]P or its active metabolite BPDE. Transcriptional response to BPDE was comparable in both cell types. However, since B[a]P will be metabolized to BPDE only in +*Ahr*, but not in -*Ahr* cells, no differential expression of these genes was detectable in -*Ahr* cells for B[a]P exposure, while in +*Ahr* cells differential expression was observed with a time lag (Δt) compared to exposure to the BPDE itself.

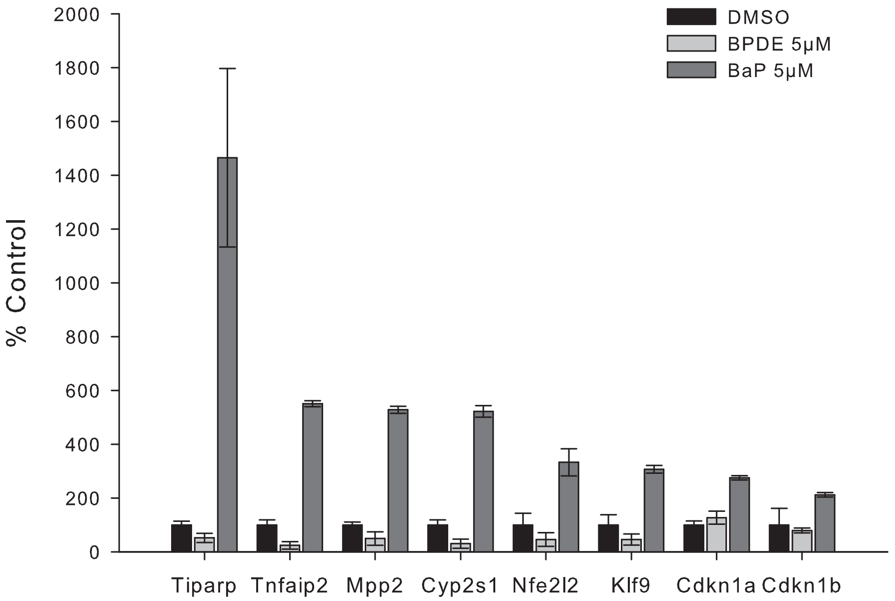

Figure S 9: Primary *Ahr* target genes are not activated by BPDE. Cells were exposed for 2h to 5μM B[a]P or BPDE respectively. No significant upregulation of predicted primary *Ahr* targets by BPDE was observed.

ID	Term	Annotated	Significant	Expected	p
GO:0051028	mRNA transport	54	18	5.99	1.1e-05
GO:0051301	cell division	264	57	29.30	2.6e-05
GO:0001701	in utero embryonic development	213	44	23.64	3.3e-05
GO:0007050	cell cycle arrest	54	17	5.99	4.6e-05
GO:0043065	positive regulation of apoptosis	265	57	29.41	4.6e-05
GO:0008285	negative regulation of cell proliferatio...	212	43	23.53	6.3e-05
GO:0000059	protein import into nucleus, docking	15	8	1.66	7.1e-05
GO:0032318	regulation of Ras GTPase activity	78	21	8.66	8.3e-05
GO:0045944	positive regulation of transcription fro...	345	62	38.29	8.4e-05
GO:0006468	protein amino acid phosphorylation	802	131	89.01	9.6e-05
GO:0007169	transmembrane receptor protein tyrosine ...	212	48	23.53	0.00011
GO:0051130	positive regulation of cellular componen...	111	26	12.32	0.00016
GO:0051726	regulation of cell cycle	269	50	29.86	0.00017
GO:0043066	negative regulation of apoptosis	241	52	26.75	0.00017
GO:0006511	ubiquitin-dependent protein catabolic pr...	156	33	17.31	0.00019
GO:0001525	angiogenesis	159	33	17.65	0.00027
GO:0007067	mitosis	205	48	22.75	0.00028
GO:0016477	cell migration	298	52	33.07	0.00062
GO:0046777	protein amino acid autophosphorylation	67	17	7.44	0.00081
GO:0015813	L-glutamate transport	20	8	2.22	0.00083
GO:0006309	DNA fragmentation involved in apoptosis	12	6	1.33	0.00095
GO:0008286	insulin receptor signaling pathway	40	12	4.44	0.00096
GO:0006915	apoptosis	812	156	90.12	0.00099

Table S 2: Enrichement of GO biological processes among 2,338 DE genes (with an enrichment p value of less than 0.001).

cluster	ID	Term	Annotated	Significant	Expected	p
1	GO:0051301	cell division	35	32	21.83	9.1e-05
1	GO:0019941	modification-dependent protein catabolic...	57	47	35.55	0.00064
1	GO:0006468	protein amino acid phosphorylation	81	63	50.52	0.00162
1	GO:0006260	DNA replication	13	13	8.11	0.00207
1	GO:0007067	mitosis	30	26	18.71	0.00299
1	GO:0016568	chromatin modification	26	23	16.22	0.00301
1	GO:0065002	intracellular protein transmembrane tran...	12	12	7.48	0.00334
1	GO:0016043	cellular component organization	202	155	125.99	0.00678
1	GO:0007265	Ras protein signal transduction	27	23	16.84	0.00841
1	GO:0006606	protein import into nucleus	10	10	6.24	0.00869
1	GO:0051128	regulation of cellular component organiz...	34	28	21.21	0.00924
2	GO:0007186	G-protein coupled receptor protein signa...	71	40	22.04	4.3e-06
2	GO:0015672	monovalent inorganic cation transport	19	12	5.90	0.0036
2	GO:0006952	defense response	30	16	9.31	0.0083
3	GO:0050793	regulation of developmental process	104	16	6.84	0.00064
3	GO:0042221	response to chemical stimulus	82	17	5.40	0.00070
3	GO:0010033	response to organic substance	42	9	2.76	0.00107
3	GO:0043086	negative regulation of catalytic activit...	14	5	0.92	0.00135
3	GO:0055114	oxidation reduction	54	10	3.55	0.00184
3	GO:0048522	positive regulation of cellular process	129	17	8.49	0.00262
3	GO:0010817	regulation of hormone levels	10	4	0.66	0.00268
3	GO:0008285	negative regulation of cell proliferatio...	24	6	1.58	0.00343
3	GO:0007050	cell cycle arrest	11	4	0.72	0.00400
3	GO:0046942	carboxylic acid transport	12	4	0.79	0.00570
3	GO:0032879	regulation of localization	36	7	2.37	0.00709
3	GO:0070887	cellular response to chemical stimulus	20	5	1.32	0.00763
3	GO:0006974	response to DNA damage stimulus	28	6	1.84	0.00777
3	GO:0006869	lipid transport	13	4	0.86	0.00783
3	GO:0048514	blood vessel morphogenesis	29	6	1.91	0.00929

Table S 3: Enrichment of clusters for GO biological processes. Enrichment was calculated against the pooled annotations of all genes assigned to any of the three clusters (i.e. not against genome-wide annotations).

gene	forward primer	reverse primer
Tiparp	TTGGAAATTCTTCTGTAGAGACCAC	CTTCTTCAATTAGTCGAACAACAGAC
Tnfaip2	ACGTGGGGATGCAGATAAAG	GCTCTTCTCTAGAAACTCATCAAAGG
Cdkn1b	TTGGGTCTCAGGCAAACTCT	TTGGGTCTCAGGCAAACTCT
Nfe2l2	GCAGCCATGACTGATTTAAGC	TAGCTCCTGCCAAACTTGCT
Cdkn1a	AACATCTCAGGGCCGAAA	TGCGCTTGGAGTGATAGAAA
Mpp2	GCGTGTCCACTAAGCAGTTG	GGCTGCTCTCCTCCACTGT
Cyp2s1	TGCTGAGATACCCTCAAGTCC	CAGGACCCAGCTCCTGTATG
Klf9	GAGAGCTTGATGTTCCAGCA	AGGAGGCAGGTTCATTTGAG
Nfkb	AGGGAGTGGTGCCAGGTA	CGGTTTCCCATTTAGTATGTCAA
Adora1	TCCTCACCCAGAGCTCCAT	GAGTCACCACTGTCTTGTACCG
Anapc	CAACATGGCGGCTCTAAGTC	CGCCCCTGAGAAAGAGTG
Parp1	CAAGGGCTTTAGCCTCCTCT	CATCCACCTCGTCACCTTTT
Myst2	GAGTCACCCGCTCCTCAG	TTTCGAACAGGGCTGGAAT
Axin2	GAGAGTGAGCGGCAGAGC	CGGCTGACTCGTTCTCCT
Lig3	CCAGTACCAATCCTCGGAAG	TTGCTCAGAGTTGTTGGGTTT
Gapdh	TGTGTCCGTCGTGGATCTGA	CCTGCTTCACCACCTTCTTGA
18S rRNA	CGGCTACCACATCCAAGGAA	GCTGGAATTACCGCGGCT

Table S 4: PCR primers for qPCR gene expression validation experiments.

gene	forward primer	reverse primer
Tiparp	TTGCCTGGATTGGTGTGATA	AGGCTCAGTTGGCACAGATT
Tnfaip2	CGTGAGATGGGTCTTGGACT	TGTTTTTCCTGGCCCTACTG
Cdkn1b	GCCGTTTGGCTAGTTTGTTT	GAGGTGTACGACTGCCAACA
Nfe2l2	GCCCAGGGGAAAATAACAAT	GGGACAAGTTGGAGCTGTTG
Cdkn1a	TCTGGTTTCCCAACATAGGC	CAAAACACGTCACGATGAGC
Mpp2	CCCCTCTGTCAACTCCATGT	GAGCAGACCCAGGCTTACTG
Cyp2s1	CTCCTGCCTCCCTCTGCTTA	CGGAGCCTTGTAATCTAGGG
Klf9	GAGAGCTTGATGTTCCAGCA	AGGAGGCAGGTTCATTTGAG
Gapdh	TGGGTGGAGTGTCCTTTATCC	TATGCCCGAGGACAATAAGG

Table S 5: PCR primers for ChIP experiments.
